# A Low-Cost and Lithium-Free Hole Transport Layer for Efficient and Stable Normal Perovskite Solar Cells

**DOI:** 10.3390/nano13050883

**Published:** 2023-02-26

**Authors:** Nikolaos Tzoganakis, Dimitris Tsikritzis, Konstantinos Chatzimanolis, Xiaodong Zhuang, Emmanuel Kymakis

**Affiliations:** 1 Department of Electrical & Computer Engineering, Hellenic Mediterranean University (HMU), 71410 Heraklion, Crete, Greece; 2 Institute of Emerging Technologies (i-EMERGE) of HMU Research Center, 71410 Heraklion, Crete, Greece; 3 Meso-Entropy Matter Lab, State Key Laboratory of Metal Matrix Composites Shangai Key Laboratory of Electrical Insulation and Thermal Gaining, School of Chemistry and Chemical Engineering, Frontiers Science Center for Transformative Molecules, Shanghai Jiao Tong University, Shanghai 200240, China

**Keywords:** perovskite solar cell, hole transport layer, X60, Li-TFSI, EMIM-TFSI, stability

## Abstract

The most widely used material as a hole-transport layer (HTL) for effective normal perovskite solar cells (PSCs) is still 2,2′,7,7′-Tetrakis[N, N-di(4-methoxyphenyl)amino]-9,9′-spirobifluorene (Spiro-OMeTAD), which requires heavy doping with the hydroscopic Lithium bis(trifluoromethanesulfonyl)imide (Li-ΤFSI). However, the long-term stability and performance of PCSs are frequently hampered by the residual insoluble dopants in the HTL, Li^+^ diffusion throughout the device, dopant by-products, and the hygroscopic nature of Li-TFSI. Due to the high cost of Spiro-OMeTAD, alternative efficient low-cost HTLs, such as octakis(4-methoxyphenyl)spiro[fluorene-9,9′-xanthene]-2,2′,7,7′-tetraamine) (X60), have attracted attention. However, they require doping with Li-TFSI, and the devices develop the same Li-TFSI-derived problems. Here, we propose Li-free 1-Ethyl-3-methylimidazolium bis(trifluoromethanesulfonyl)imide (EMIM-TFSI) as an efficient p-type dopant of X60, resulting in a high-quality HTL with enhanced conductivity and deeper energy levels The optimized X60:EMIM-TFSI-enabled devices exhibit a higher efficiency of 21.85% and improved stability, compared to the Li-TFSI-doped X60 devices. The stability of the optimized EMIM-TFSI-doped PSCs is greatly improved, and after 1200 hr of storage under ambient conditions, the resulting PSCs maintain 85% of the initial PCE. These findings offer a fresh method for doping the cost effective X60 as the HTL with a Li-free alternative dopant for efficient, cheaper, and reliable planar PSCs.

## 1. Introduction

Over the last decade, perovskite solar cells (PSCs) have monopolized the interest of the scientific community as they have reached a power conversion efficiency (PCE) of 25.7% [1]. However, despite their excellent performance and low production cost, the long-term stability and durability against environmental factors, and the presence of a number of defects, are barriers on the road to commercialization [2,3,4]. PSCs are exhibiting excellent results in terms of efficiency compared to those of Si and CIGS solar cells [5,6,7]. The most efficient PSCs employ the n-i-p device architecture and incorporate 2,2′,7,7′-Tetrakis[N,N-di(4-methoxyphenyl)amino]-9,9′-spirobifluorene (Spiro-OMeTAD) as the hole transport layer (HTL), which must be doped with various additives, the most well-known being Lithium bis(trifluoromethanesulfonyl)imide (Li-ΤFSI) and 4-tert-Butylpyridine (t-BP) [5,6,7]. Spiro-OMeTAD suffers from low conductivity and unfavorable energy levels for perovskite devices, and in order to increase the hole mobility and to tune the energy levels of Spiro-OMeTAD, various p-dopants are employed. As well as Li-TFSI, Tris(pentafluorophenyl)borane (BCF), 2,3,5,6-tetrafluoro-7,7,8,8-tetracyanoquinodimethane (F4TCNQ), Co (III) complexes (FK102, FK209, FK269, etc.), molybdenum complexes (Mo(tfd-COCF_3_)_3_ Mo(tfd-CO_2_Me)_3_), SnCl_4_, and various copper salts (CuSCN, CuI) have been reported as p-type dopants [8,9,10,11,12,13,14,15]. However, most of these p-type dopants require laborious purification procedures and multistep synthetic routes. More importantly, many reports emphasize the negative effects of these dopants on device performance and stability. For example, oxygen and moisture promote the diffusion of additives, altering the electrical properties and morphology of the HTL. Although t-BP is used to increase the solubility of Li-TFSI in the HTL, it has been shown that, over time, it diffuses to the perovskite layer and promotes degradation. Heating the device during operation modifies the work function energy levels of the doped Spiro-OMeTAD, affecting the transport of holes from the perovskite [16,17,18].

The complicated synthetic procedure of Spiro-OMeTAD increases the production cost, and the high cost of Spiro-OMeTAD is a significant disadvantage for large-area PSCs. In addition, the requirement of heavy doping with Li-TFSI complicates the fabrication procedure while inducing dopant-related problems. Therefore, numerous small molecules and polymers have been developed for the HTL, as alternatives to Spiro-OMeTAD, including derivatives of triphenylamine, thiophene, and phthalocyanine [3,19,20,21], but only few exhibit efficiency more than 20% [22,23,24]. Additionally, many of these materials have low solubility in common polar solvents, poor film morphology, low mobility, and stability issues. When these materials are utilized in PSCs, the devices exhibit poor performance and stability and require a high concentration of dopants. Furthermore, the unreacted residuals and byproducts that remain in the doped HTL film are frequently detrimental to the PCE and stability of the PSCs [25]. Therefore, it is crucial to investigate new, inexpensive materials in order to create effective and reliable perovskite photovoltaics.

Ionic liquids, which are essentially a subset of molten salts, have recently been investigated for use in PSCs, and significant work has been accomplished in this area. Typically, they contain a variety of organic cations (such as imidazolium, pyrrolidinium, triazolium, pyridinium, piperidinium, and phosphonium) as well as organic or inorganic anions (such as phosphate, carboxylic acid, sulfonic acid, imide, and halide). In general, ILs exhibit a variety of special qualities, including low toxicity, solvate capability, a broad liquid range, high conductivity, and a broad electrochemical window. According to reported studies, ILs can perform a variety of tasks during the manufacture and operation of PSCs, depending on the choice of specific chemical structures. ILs are utilized in PSCs in three categories: (a) improving charge-transport layers by adding dopants to hole transport materials (HTMs) or electron transport materials (ETMs), (b) enabling thin-film manufacturing of perovskites, and (c) enhancing interfacial energetics between perovskite and other layers [26,27,28,29,30,31,32,33,34,35].

Most reported alternative Spiro-OMeTAD and high performance HTLs were designed based on the similar Spiro-OMeTAD chemical structure, Spiro(fluorene-9,9′-xanthene) (SFX) basic structure, which includes fluorene and xanthene units. These chemical units improve the thermal and oxidative stability of the material [36,37]. The SFX-based materials can be synthesized with low cost, while the oxygen atoms in the structure improve the solubility and the morphological properties of the material and tune the optical properties [22]. Perovskite devices incorporating SFX-based HTLs were fabricated, delivering PCE near 20% and good stability [24,38,39,40]. Among the investigated SFX-based HTLs, the commercially available octakis(4-methoxyphenyl)spiro[fluorene-9,9′-xanthene]-2,2′,7,7′-tetraamine) (X60) stands out due to its low cost and high performance in PSCs [41,42,43]. A detailed steady-state and time-resolved spectroscopic study revealed that X60 forms an excellent interfacial contact with perovskite, providing very fast hole transport at the interface. Compared to Spiro-OMeTAD, X60 costs 30 times less, due to its one-pot synthesis. However, the X60 suffers from low hole conductivity and is typically doped with Li-TFSI [44,45,46]. The PSCs that use X60 as the HTL exhibit high efficiency and are compatible with devices containing Spiro-OMeTAD but show the same Li-TFSI-related problems [4]. To eliminate the Li^+^ related problems, the ionic liquid dopant BuPyIm-TFSI was used with X60. However, the resulting devices exhibited limited performance up to 14.65% [47]. Inspired by the good performance of X60 in PSCs, we used it as the HTL, and we endeavored for high performance devices without Li-TFSI as a dopant. The aim of this work is to show a cheaper alternative to Spiro-OMeTAD, exhibiting PCE greater than 21% and improved stability, without using the problematic Li-TFSI but with an effective Li-free dopant. The EMIM-TFSI can oxidize X60 efficiently, increasing the conductivity by two orders of magnitude and lowering the energy levels of X60 by about 0.3 eV. The EMIM-TFSI-doped X60 exhibits better energy level alignment with the perovskite and the top metal electrode and thus better performance.

## 2. Materials and Methods

### 2.1. Materials

All chemicals were used as received without further purification. SnO_2_ 15% in H_2_O colloidal dispersion (Alfa Aesar, Haverhill, MA, USA), deionized water (Sigma Aldrich, St. Louis, MI, USA), perovskite precursor: formamidinium iodide (FAI, 99.995%, GreatCell Solar, Queanbeyan, Australia), Methylammonium Iodide (MAI, 99.995% GreatCell Solar), methylammonium chloride (MACl, 98% GreatCell Solar), benzamidine hydrochloride (Ph-FACl, 99.95%, Sigma Aldrich), PbI_2_ (99.99%, TCI Japan, Tokyo, Japan), dimethylformamide (DMF, 99.8%, Acros, Geel, Belgium), dimethyl sulfoxide (DMSO, 99.9%, Acros), isopropanol (IPA, 99.5%, Acros Organics), X60 (99.8%, Dyen-amo, Stockholm, Sweden), 4-tert-butylpyridine (tBP, 96%, Sigma Aldrich), Li-bis(trifluoromethanesulfonyl) imide (Li-TFSI, 99.95%, Sigma Aldrich), Acetonitrile (ACN, 99.8%, Acros Organics), Chlorobenzene (CB, 99.8%, Acros Organics), 1-Ethyl-3-methylimidazolium bis(trifluoromethanesulfonyl)imide (EMIM-TFSI, 98%, TCI America, Portland, OR, USA), MoO_3_ (99.99%, J.K Lesker, Jefferson Hills, PA, USA), Ag (99.999%, J.K Lesker).

### 2.2. Device Fabrication

A sequence of washes with distilled water, acetone, and isopropanol were used to clean the ITO substrate (Naranjo). After coating the ITO substrate with SnO_2_ (diluted to 2.47 wt% in deionized water), the layer was spin coated at 6000 rpm for 45 s and subsequently annealed in air at 150 °C for 30 min to form the electron transport layer. The substrate was brought down to room temperature, treated with ultraviolet ozone for 10 min, and then coated with a PbI_2_ solution. The PbI_2_ powder was dissolved in DMF/DMSO (*v*/*v*, 94/6) at a concentration of 1.5 M and stirred at 70 °C for 15 h. FAPbI_3_ perovskite was prepared by a two-step process. First, the PbI_2_ solution was deposited by spin coating at 1900 rpm for 30 s, dried at 70 °C for 1 min, and then cooled to room temperature. On top of the PbI_2_ layer, a solution of FAI/MAI/MACl/Ph-FACl (76.5:13.5:18:3.5 mg) in 1 mL of isopropanol was spin-coated at a rotation speed of 2500 rpm for 40 s [48,49]. This was followed by thermal annealing at 160 °C for 10 min in ambient conditions (approximately 40% RH). The HTL solution was then spin-coated at 3000 rpm for 30 s. The HTL for the control devices was prepared by dissolving 80 mg of X60, 30 μL of 4-tert-butylpyridine (tBP), and 35 μL of 260 mg mL^−1^ Li-TFSI in acetonitrile in 1 mL chlorobenzene. For the EMIM-TFSI-doped X60 devices, various percentages (wt%) of EMIM-TFSI (4%, 8%, 12%, 16%) were added to the X60 solution instead of Li-TFSI, with the molar ratio of tBP remaining constant. The as-deposited incomplete devices were aged for ∼12 h under dry air conditions (30% RH). Finally, a 15 nm MoO_x_ layer and a 80 nm Ag layer were deposited by thermal evaporation under a pressure of 1.0 × 10^−4^ mbar. The effective area was 0.04 cm^2^, as defined by an aperture mask.

### 2.3. Device Characterization

The FS5 spectrofluorometer was used to measure steady-state photoluminescence (PL) (Edinburgh Instruments, Livingston, UK). The work function (WF) and the higher occupied molecular orbital (HOMO) level were estimated by ambient photoemission spectroscopy (APS) using an APS04 N2-RH setup (KP Technology, Wick, UK). Specifically, a vibrating gold alloy probe was used to assess the contact potential difference (CPD) (2 mm in diameter). The absolute WF of a polished silver reference was measured, and its WF was determined using APS to calibrate the tip WF, which was calculated to be 4.54 eV. By extrapolating the cube root of the photoemission signal to zero and using a UV light excitation source (D2) with an excitation energy in the region of 3.8–6 eV, HOMO was obtained. The bandgap of Perovskite was calculated by the 1st derivative of the EQE spectrum. The LUMO level of X60 was calculated by adding the HOMO values as estimated from APS to the optical bandgap provided from the supplier (Dyenamo) using the following formula:

(1) $$E_{\mathrm{LUMO}}=E_{\mathrm{HOMO}}+E_{g}$$

where E_g_ is the bandgap. The small-perturbation regime was used for the operation of the transient photovoltage by setting the maximum amplitude of the LED pulse to 10% of the equilibrium voltage established at the background field (Arkeo, Cicci Research s.r.l., Grosseto, Italy). This method ensured that the transient voltage signal dropped linearly, and the resulting fits reflected the lifespan of the charge carriers. Additionally, this method assured that the fit values were accurate representations of the lifetime of the charge carriers. The transient photocurrent response, on the contrary, was monitored under conditions of large perturbation and 50 m extraction to an external circuit. The duty cycle was adjusted to 1/2 in order to give sufficient time for device loading and decay tail relaxation. The responses of the transimpedance amplifier and the differential voltage amplifier were used in order to manage the open circuit voltage and the short circuit current circumstances. For trap density measurements, the trap density of hole-only devices was measured by the space-charge-limited-current (SCLC) method using a diode configuration of ITO/PTAA/Perovskite/doped X60/Au. The J–V characteristics of the devices were acquired using a solar simulator (Oriel, Darmstadt, Germany) equipped with a 450 W Xenon lamp. The intensity of the lamp was calibrated at 100 mW cm^−2^ (1 Sun) AM1.5G illumination using a Keithley 2700 data acquisition system equipped with a KG-5 Si diode. The J–V curves of the devices were measured in an N_2_ filled glovebox (MBRAUN, Garching, Germany) with a multiplexor test board system (Ossila, Sheffield, UK) by scanning from 0.1V to 1.2 V, with a scan rate of 10 mV s^−1^ and a voltage step of 10 mV. A metal mask was used to define the active area of the solar cells (0.04 cm²). External quantum efficiency (EQE) was measured with a commercial system from Enlitech (QE-R2 Kaohsiung City, Taiwan) with a chopping frequency of 65 Hz. The thicknesses were estimated by a stylus profilometer (DektakXT, Bruker, Leidedorp, The Netherlands).

## 3. Results

**Figure 1:**
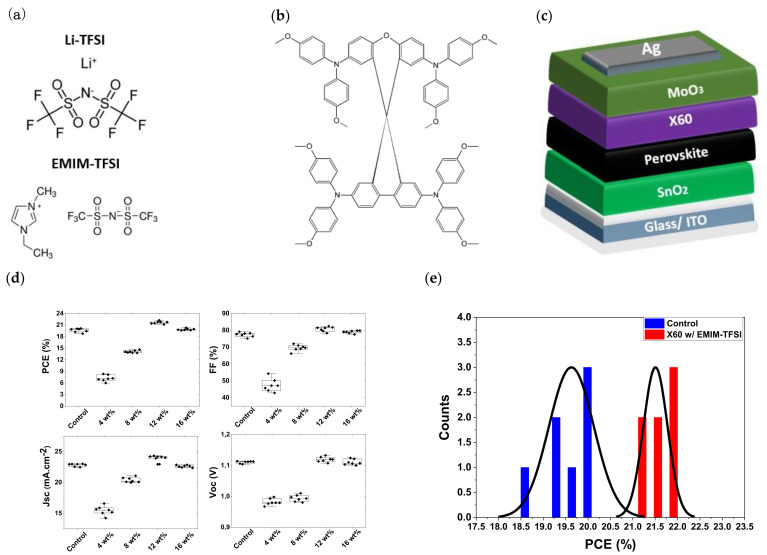
Molecular structures of Li-TFSI and EMIM-TFSI in (**a**) and X60 in (**b**). (**c**) Schematic structure of the planar perovskite solar cell manufactured in this work (not in scale). (**d**) Evolution of photovoltaic parameters with increasing EMIM-TFSΙ concentration. (**e**) Statistic distribution of PCE of control and 12% EMIM-TFSI-doped devices.

**Figure 2:**
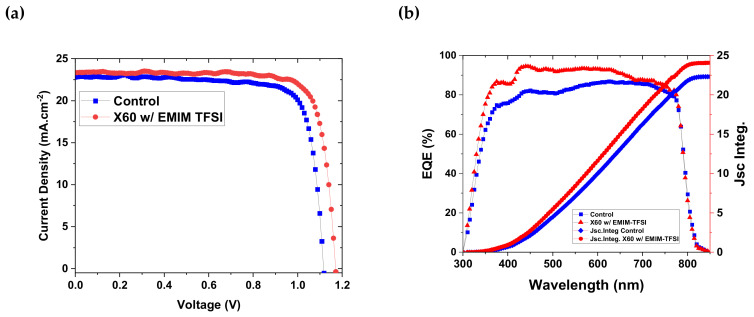
(**a**) J–V curves of the best performing control and EMIM-TFSI-doped (12 wt%) devices. (**b**) EQE spectra for the best performing devices. The integrated current density depicted on the right axis was calculated from the device’s spectral response and the AM1.5G photon flux spectrum. Integrated current density was estimated at 22.85 and 23.97 and mA cm^−2^ for the control and EMIM-TFSI (12 wt%), respectively.

The chemical structures of the Li-TFSI and EMIM-TFSI dopants are depicted in Figure 1a. The EMIM-TFSI contains an imidazolium cation instead of the Li^+^ of Li-TFSI. Li^+^ is known to diffuse through the PSC layers and to cause stability and performance issues [50]. Therefore, an efficient dopant of the HTL without the Li^+^ cation is highly desirable. To assess the direct impact of EMIM-TFSI on the photovoltaic performance, different percentages of EMIM-TFSI were added to the X60 solution, and PSCs were fabricated with EMIM-TFSI-doped X60 as the HTL. The molecular structure of X60 and the planar device structure used in this work are depicted in Figure 1b,c respectively. Statistical findings from the same batch of ten devices are shown in Figure 1c. The control devices contain X60 doped with Li-TFSI. Compared to the control devices, all photovoltaic parameters, i.e., open circuit voltage (V_oc_), short circuit current (J_sc_), fill factor (FF), and PCE improve with increasing concentration of EMIM-TFSI, reaching an optimal performance at 12 wt% and an increased average PCE by +9.1%. In particular, the V_oc_ significantly increases, which is most likely a result of the dopant’s higher WF and surface passivation effect. The PSCs based on X60:EMIM-TFSI with an optimal doping percentage of 12 wt% exhibited the best PCE performance of 21.85%, with a high FF of 0.807, Voc of 1.132 V, and Jsc of 23.90 mA/cm^2^. This performance is comparable to that of devices that incorporate Spiro-OMeTAD as HTL doped with Li-TFSI and Co-TFSI [7,51,52]. The statistical distribution of the best performing devices is depicted in Figure 1d, which attests to the excellent repeatability of the devices. Moreover, in Table 1 the mean and champion photovoltaic parameters are listed. The photovoltaic parameters were extracted from the corresponding J–V curves. Figure 2a shows the reverse J–V scans of the control and best performing EMIM-TFSI devices. The hysteresis index can be calculated using the formula HI = (PCE_(RS)_ − PCE_(FS)_)/PCE_(RS)_, where PCE_(RS)_ and PCE_(FS)_ represent the reverse and forward scans, respectively (HI). According to Table 2, both PSCs with X60:Li-TFSI and X60:EMIM-TFSI exhibit low HI values of 0.051 and 0.027, respectively. As we will show later, the decreased defect density at the perovskite/HTL interface and the absence of the Li^+^ cation in the device may be the cause of the hysteresis reduction observed in the EMIM-TFSI-containing devices. The EQE spectra for the control and best performing PSC are shown in Figure 2b. The integrated current densities obtained in the range of 300–850 nm were calculated to be 22.85 and 23.97 mA.cm^−2^ for the control and optimal-doped devices with EMIM-TFSI, respectively (Figure 2b).

**Figure 3:**
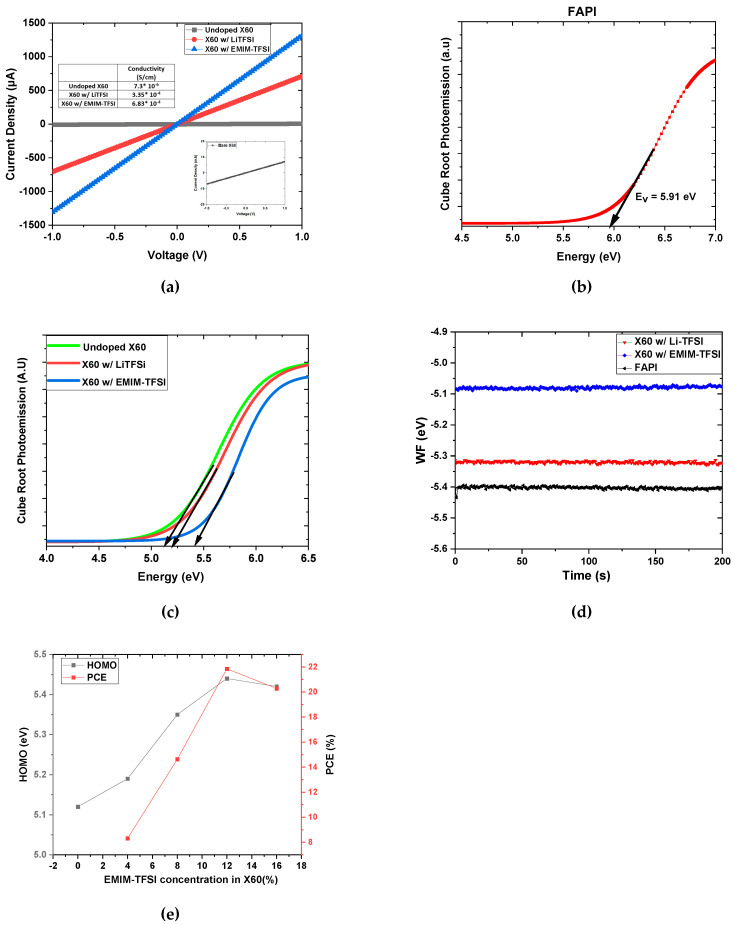
(**a**) Two-probe conductivity characterization. Cube root photoemission response of (**b**) FAPI and (**c**) undoped X60, LiTFSi, and EMIM-TFSI-doped X60. (**d**) WF measurements of PVSK and HTLs in the dark, and (**e**) the HOMO energy level position of X60 and the PCE as a function of EMIM-TFSI concentration.

**Figure 4:**
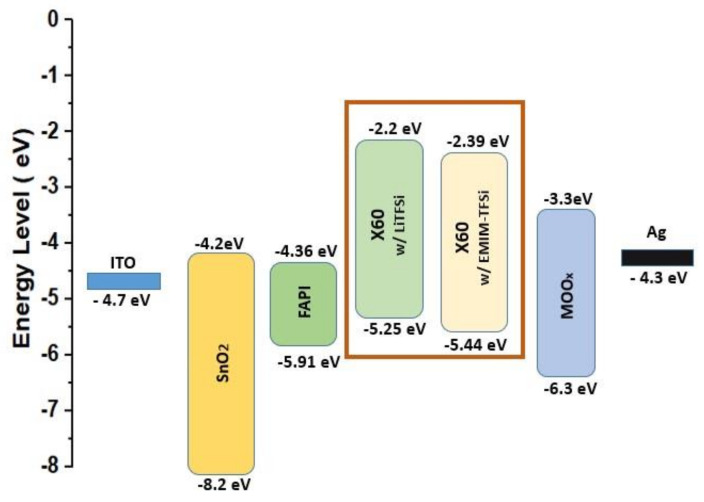
Energy level alignment in the device stack. In the figure are shown the energy levels of X60 doped with Li-TFSI and with EMIM-TFSI. Doping with EMIM-TFSI lowers the energy levels of X60.

A crucial electrical property that affects the performance of HTLs is conductivity (σ), which can be estimated through direct current–voltage (I–V) measurements. We adopted the ITO/X60/Ag design for the I–V measurements. The conductivity can be determined from the following equation.

(2) $$\sigma =\frac{Id}{\mathrm{VA}}$$

where A is the area of the electrodes (0.04 cm^2^), d is the thickness of the X60 layer (200 nm), V is the bias that was applied, and I is the real-time current. The acquired I–V curves are shown in Figure 3a, and the slope of the linear curve gives the conductivity. Therefore, σ was estimated at 7.3 × 10^−6^ S/cm, 3.35 × 10^−4^ S/cm, and 6.83 × 10^−4^ S/cm for X60: dopant-free, X60:Li-TFSI, and X60:EMIM-TFSI, respectively. The undoped X60 shows low conductivity but increases by two orders of magnitude upon doping with Li-TFSI and EMIM-TFSI, with the EMIM-TFSI-doped X60 exhibiting the highest electrical conductivity. The high electric conductivity of EMIM-TFSI-doped X60 indicates that the former readily oxidizes X60, p-type doping it [53,54]. To determine the energy levels of X60, APS and dark work function measurements were conducted. The valence band maximum (VBM) of the perovskite and the HOMO of X60 were estimated from the spectra demonstrated in Figure 3b,c. The undoped X60 shows a HOMO at −5.12 eV, which decreases to −5.25 eV upon Li-TFSI doping. However, when X60 is doped with EMIM-TFSI, the HOMO of X60 decreases to −5.44 eV, getting closer to the VBM of perovskite at −5.91 eV. This energy level adjustment with a reduced energy level difference between the HOMO of HTL and VBM of perovskite enhances the hole transport at the perovskite/HTL interface [55]. Figure 3e shows the correlation of the HOMO energy level position of X60 with PCE. With increasing EMIM-TFSI concentration, the HOMO has lower values, and the PCE increases, reaching a maximum at 12%. A higher doping concentration slightly increases the HOMO, and the PCE declines. Additionally, the X60:EMIM-TFSI has a higher WF value (5.32 eV) than X60:Li-TFSI (5.09 eV), as depicted in Figure 3d, which aligns better with the WF of MoO_x_ and facilitates hole transport. Figure 4 shows the corresponding schematic energy level diagram. As is well known, the difference in Fermi Level (E_F_) between the electron transport materials (ETMs) and the hole transport materials (HTMs) in PSCs determines the device’s V_oc_. Therefore, it is assumed that X60:EMIM-TFSi-based devices with deeper WF will display higher V_oc_ in PSCs. Moreover, the V_oc_ is affected by the not-radiative recombination in the bulk and at the surfaces [56,57,58]. The enhanced performance and higher V_oc_ of the X60:EMIM-TFSI devices can be related to reduced non-radiative recombination. As will be shown later in the manuscript with additional measurements, the X60:EMIM-TFSI devices exhibit reduced trap density and enhanced built-in potential, indicating reduced nonradiative recombination in the devices.

**Figure 5:**
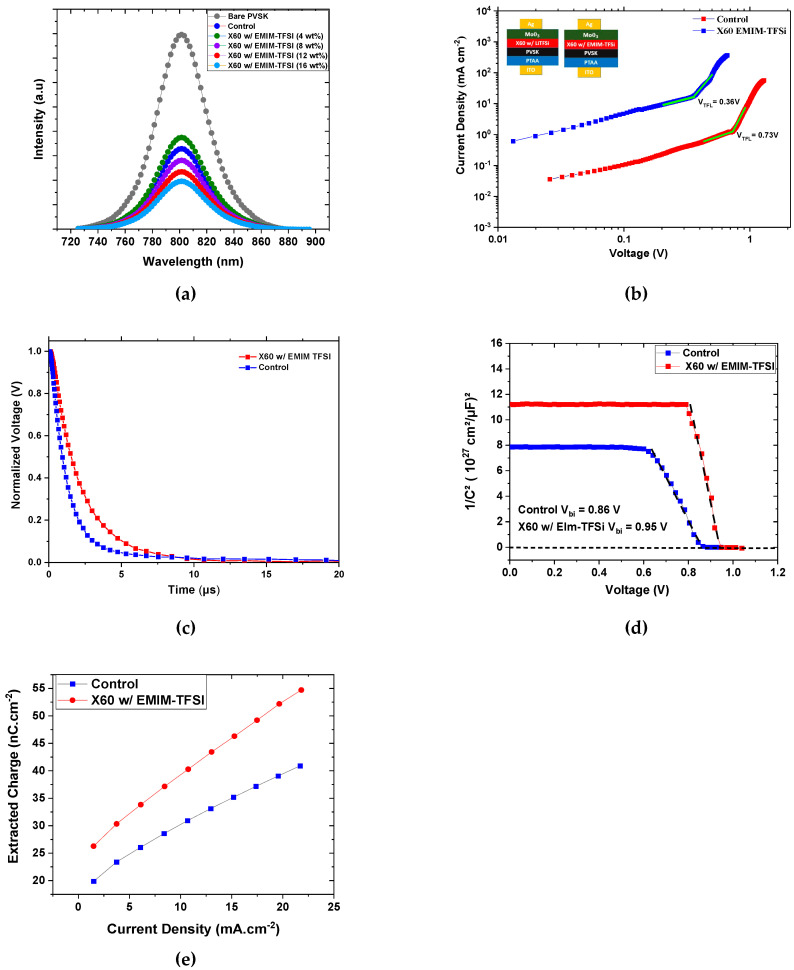
(**a**) Steady-state photoluminescence of ITO/perovskite samples without HTL, covered with X60 doped with Li-TFSI and covered with X60 doped with EMIM-TFSI (12 wt%). Characterization of control and EMIM-TFSI-doped X60 devices: (**b**) dark J–V curves of hole only devices, (**c**) transient photovoltage (TPV) curves, (**d**) Mott–Schottky analysis, and (**e**) extracted charge estimation from TPC measurements for the completed best performing devices.

The previous results show that EMIM-TFSI has the ability to act as an efficient dopant to control interfacial energy levels and X60 oxidation. The PL spectra in Figure 5a support the superior hole extraction capabilities of the EMIM-TFSI-doped X60. Specifically, the quenching of the PL intensity of the ITO/perovskite/X60:EMIM-TFSI sample indicates faster hole extraction compared to the control. The quenching of the PL spectra follows the concentration of EMIM-TFSI, that is, higher dopant concentration comes with reduced PL intensity. The PL results correlate with the improved PCE upon doping with EMIM-TFSI, and a concentration of 12% is needed for optimal performance. Although the doping with 16% induces further PL quenching, the PCE does not increase further due to the none-optimized energy level alignment at the perovskite/HTL interface for high doping concentration, as we have shown previously. Figure 5b depicts the dark J–V scans of hole-only devices exhibiting the trap-filled limit voltage (VTFL) characteristic. The following equation, which considers the elementary charge (1.6 × 10^−19^ C), the trap-state density (N_t_), the thickness of the perovskite film (L), estimated to be 450 nm, the relative dielectric constant (ε) (30 for perovskite), and the vacuum permittivity (ε_ο_), was used to estimate the trap density [59].

(3) $$N_{t}=\frac{2{\varepsilon \varepsilon }_{0}V_{\mathrm{TFL}}}{{\mathrm{qL}}^{2}}$$

The trap density was calculated at 1.20 × 10^16^ cm^3^ and 5.90 × 10^15^ cm^3^ for the Li-TFSI and EMIM-TFSI-doped X60 devices, respectively. Interestingly, the EMIM-TFSI-doped devices exhibited lower trap density compared to the control, and consequently the trap assisted nonradiative recombination must have been reduced. Indeed, the optimized EMIM-TFSI-doped devices deliver higher V_oc_, which is the result of reduced non-radiative recombination in the device. In addition, the degeneration of the transient photovoltage (TPV) was investigated, as shown in Figure 5c. All photogenerated charge carriers will recombine when the device is maintained in the open-circuit condition; as a result, the recombination process will reflect the device’s characteristics. It is abundantly clear that the charge recombination lifetime of the device based on X60:EMIM-TFSI is longer than that of the device based on X60:Li-TFSI, which is in agreement with the increased Voc. These results indicate that the non-radiative pathway of the EMIM-TFSI-doped HTL was reduced, and the charge extraction was improved, which is compatible with the PL results. Additionally, the non-radiative pathway of the EMIM-TFSI-doped HTL was reduced. The built-in potential (V_bi_) of the two devices was estimated following Mott–Schottky analysis, as illustrated in Figure 5d. The following equation describes the relationship between capacitance and DC voltage bias [60]:

(4) $$\frac{1}{C^{2}}=\frac{2\left(V_{\mathrm{bi}}-V\right)}{{q\varepsilon \varepsilon }_{0}A^{2}N}$$

where V represents the applied DC bias, N denotes the impurity doping density, A denotes the active area of the device, and ε_ο_ and ε_r_ represent vacuum and relative permittivity, respectively. The Vbi can be determined using the X-intercept of the linear regime of the M–S plot. The device with EMIM-TFSI-doped X60 exhibited a higher V_bi_ of 0.96 V than the control device’s 0.88 V, which is consistent with the higher V_oc_ of the EMIM-TFSI-doped devices. The enhanced V_bi_ can be attributed to the reduction of nonradiative recombination due to the efficient passivation of charge defects at the perovskite surface and to the deeper HOMO of EMIM-TFSI-doped X60 [52]. The transient photocurrent measurements (TPC) involve the photo-excitation of the complete device with a short light pulse, slightly perturbing the otherwise constant bias illumination and creating charges out of the equilibrium. Then, after turning off the light, the perturbation charges will recombine, and after some time the current will reach equilibrium. The speed of the process is determined by charge carrier mobility, trap states, and doping levels [47]. The extracted charge from the TPC measurements on the full devices was plotted as a function of current density, as seen in Figure 5e. The EMIM-TFSI facilitates charge collection in comparison with Li-TFSI, which is in a good agreement with the non-radiative recombination lifetime extracted from the TPV measurements [61,62,63]. The analysis of the steady-state and transient measurements indicate the high capability of EMIM-TFSI to p-type-doped X60 and to improve the charge collection in the device.

### Stability

**Figure 6:**
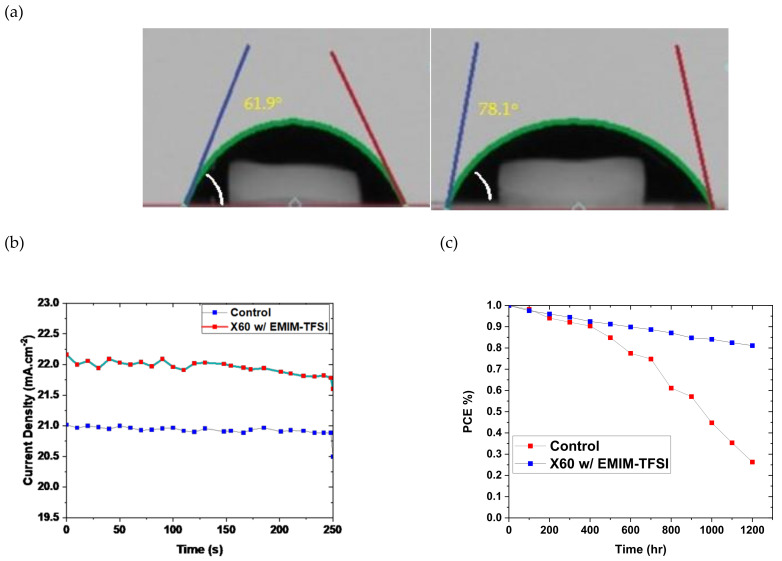
(**a**) Water contact angle measurements of X60:Li-TFSI (left) and X60:EMIM-TFSI (right). (**b**) Current density measured for 250 s at constant voltage close to the maximum power point (MPP) of the best performing devices. (**c**) Stability measurements of the unencapsulated devices according to the ISOS-D1 protocol (stored in the dark under humidity conditions of approximately 50–60% RH).

The stability of PSCs is an important performance metric for the commercial potential of PSCs. The stabilized photocurrent density at maximum power point (MPP) as a function of time is depicted in Figure 6b. Devices based on HTL doped with 12% EMI-TFSI and Li-TFSI exhibit a steady current density of 22.02 mAcm^−2^ and 21.01 mAcm^−2^, respectively, keeping in tune with the PCE values tested by J–V measurements. PSCs degrade when exposed to humidity, thermal stress, and UV light, limiting their lifetime. Numerous researchers have participated actively in efforts to improve the stability of perovskite solar cells. [56,57,58,59,60,61,62,63]. The long-term operational stability of the devices was investigated under ambient conditions, according to the ISOS D-1 protocol [60]. Unencapsulated devices were stored in the dark under ambient conditions and approximately 50–60% RH and evaluated at regular intervals. As depicted in Figure 6c, the PCE degradation curve for the X60 demonstrates that the EMIM-TFSI-based device retains approximately 88% of its initial efficiency after more than 1100 h of storage. After 950 h of operation under the same aging conditions, the PCE of devices with X60:Li-TFSI declines to 45% of its initial value. To further evaluate the stability of EMIM-TFSI-doped devices, we measured the water contact angle. Figure 6a demonstrates that the X60:EMIM-TFSI-based film has a larger contact angle (78.1°) than the Li-TFSI-doped films (61.9°). Li-TFSI is known for its high hydrophilicity and has a detrimental effect on the stability [64,65,66,67,68]. Thus, the higher hydrophobicity of X60:EMIM-TFSI shields the HTL from water penetration and hence protects the underlying perovskite from the ambient humidity. In Table 3 are listed recent articles regarding PSCs that incorporate Spiro-OMeTAD as the HTL, doped with Li-free dopants, along with devices that used X60- and SFX-based HTLs. Compared to the Spiro-OMeTAD devices, our devices exhibit similar PCEs, but better stability. Moreover, our approach delivers devices with the highest PCE among the X60-based PSCs.

## 4. Conclusions

In conclusion, we have shown that X60 is an efficient and cheaper alternative to Spiro-OMeTAD for high-performance and stable PSCs. We propose the EMIM-TFSI as an efficient dopant for the X60 HTL that provides high PCE up to 21.85%, V_oc_ of 1.132 V, increased J_sc_ of 23.90 mAcm^−2^, and an FF of 80.77, instead of the hygroscopic Li-TFSI. According to our findings, EMIM-TFSI has the potential to provide an effective route for X60 oxidation without using Li^+^ cations, which are detrimental to the performance and stability of the device. The conductivity of X60 increases by two orders of magnitude upon doping with EMIM-TFSI. Moreover, the transient and steady-state characterizations of X60:EMIM:TFI PSCs indicated reduction in the non-radiative recombination and more efficient charge extraction. The superior performance of EMIM-TFSI-doped devices is attributable to the high work function and deeper HOMO of the X60:EMIM-TFSI layer, which provide a well-matched interface energy level alignment with the perovskite valence band at the perovskite/X60 interface and WF of MoOx, hence facilitating the extraction and transport of carriers in the device. To this end, we developed an efficient oxidation method to effectively promote X60 oxidation and p-type doping using the Li-free ionic liquid 1-Ethyl-3-methylimidazolium bis(trifluoromethanesulfonyl)imide (EMIM-TFSI). The optimized devices with 12% EMIM-TFSI (mass ratio relative to X60), yield a significantly improved PCE of 21.85%. Furthermore, the unencapsulated X60:EMIM-TFSI device exhibits enhanced stability and retains 85% of its initial PCE after 1200 h of storage under ambient conditions without encapsulation due to the absence of the hygroscopic Li-TFSI in the HTL. 

## Figures and Tables

**Table 1:** The average photovoltaic parameters obtained from the J–V curves of the devices incorporating varying EMIM-TFSI weight ratios. The photovoltaic parameters of the best performing devices are placed in brackets. The errors were calculated by device statistics.

Sample	wt%	PCE (%)	FF (%)	Jsc (mA·cm^−2^)	Voc (V)
Control		19.67 ± 0.46 (20.11)	77.42 ± 1.23 (79.16)	22.82 ± 0.20 (22.97)	1.11 ± 0.03 (1.106)
X60 w/EMIM-TFSI	4%	7.19 ± 0.81 (8.31)	47.37 ± 3.83 (54.28)	15.44 ± 0.73 (15.64)	0.981 ± 0.010 (0.979)
	8%	14.09 ± 0.32 (14.63)	69.53 ± 1.86 (68.75)	20.40 ± 0.41 (21.08)	0.993 ± 0.010 (1.010)
	12%	21.65 ± 0.34 (21.85)	80.75 ± 1.45 (80.77)	23.95 ± 0.46 (23.90)	1.119 ± 0.008 (1.132)
	16%	19.85 ± 0.23 (20.28)	79.02 ± 0.82 (79.01)	22.62 ± 0.19 (22.84)	1.111 ± 0.009 (1.124)

**Table 2:** The photovoltaic parameters of the best performing devices under different scan directions and HI estimated value.

Sample	Scan Direction	PCE (%)	FF (%)	Jsc (mA·cm^−2^)	Voc (V)	HI (%)
Control	(RS) (FS)	(20.11) (19.08)	(79.16) (76.32)	(22.97) (22.86)	(1.106) (1.094)	0.051
X60 w/EMIM-TFSI (12 %wt)	(RS) (FS)	(21.85) (21.27)	(80.77) (79.65)	(23.90) (23.68)	(1.132) (1.128)	0.027

**Table 3:** Comparison table of PCE and long-term stability of normal PSCs incorporating Li-free dopants. The aging time is the duration of the lifetime measurements. PCE Loss stands for the percentage of PCE reduction at the end of the aging time.

Sample	PCE (%)	Aging Time (h)	PCE loss (%)	Reference
X60:EMIM-TFSi (Control)	21.85	1200	12	This work
X60: BuPyIm-TFSi	14.65	840	7	[47]
X60(TFSi)_2_	19.00	800	10	[43]
FDT: BMPy-TFSI	18.24	1200	10	[69]
Spiro-OMeTAD: N-Bromosuccinimide	19.24	700	12.7	[70]
Spiro-OMeTAD: CrO_3_	22.57	720	10	[71]
Spiro-OMeTAD: Er@C_82_	19.22	400	20	[72]
Spiro-OMeTAD: Sb_2_S_3_	22.13	1100	5	[73]
Spiro-OMeTAD: MWCNT:NiO	21.68	1200	10	[74]
Spiro-OMeTAD: KMnO_4_	20.03	720	11	[54]
Spiro-OMeTAD: CMP	18.72	650	15	[75]

## Data Availability

Not applicable.
